# Estrogen Attenuates Chronic Stress-Induced Cardiomyopathy by Adaptively Regulating Macrophage Polarizations via β_2_-Adrenergic Receptor Modulation

**DOI:** 10.3389/fcell.2021.737003

**Published:** 2021-09-28

**Authors:** Hongjian Hou, Gabriel Komla Adzika, Qi Wu, Tongtong Ma, Yanhong Ma, Juan Geng, Mingjin Shi, Lu Fu, Ruqayya Rizvi, Zheng Gong, Hong Sun

**Affiliations:** ^1^Department of Physiology, Xuzhou Medical University, Xuzhou, China; ^2^The College of Biology and Food, Shangqiu Normal University, Shangqiu, China; ^3^Xuzhou Medical University, Xuzhou, China; ^4^The School of Public Affairs and Governance, Silliman University, Dumaguete, Philippines

**Keywords:** chronic stress-induced cardiomyopathy, myocardial inflammation, estrogen, β2-adrenoceptors, macrophage polarization

## Abstract

Clinical demographics have demonstrated that postmenopausal women are predisposed to chronic stress-induced cardiomyopathy (CSC) and this has been associated with the decrease of estrogen. Meanwhile, recent studies have implicated unsolved myocardial proinflammatory responses, which are characterized by enormous CD86+ macrophage infiltrations as an underlying disease mechanism expediting the pathological remodeling of the heart during chronic stress. However, we had previously demonstrated that estrogen confers cardioprotection *via* the modulation of cardiomyocytes β_2_-adrenoceptors (β_2_AR)-Gs/Gi pathways during stress to lessen the incidence of stress-induced cardiovascular diseases in premenopausal women. Intriguingly, macrophages express β_2_AR profoundly as well; as such, we sought to elucidate the possibilities of estrogen modulating β_2_AR-Gs/Gi pathway to confer cardioprotection during stress *via* immunomodulation. To do this, ovariectomy (OVX) and sham operations (Sham) were performed on female Sprague-Dawley (SD) rats. Two weeks after OVX, the rats were injected with 40 μg/kg/day of estradiol (E_2_). Next, on day 36 after OVX, chronic stress was induced by a daily subcutaneous injection of 5 mg/kg/day of isoproterenol (ISO). The effect of E_2_ on relevant clinical cardiac function indexes (LVSP, LVEDP, + dp/dt and −dp/dt), myocardial architecture (cardiomyocyte diameter and fibrosis), β_2_AR alterations, and macrophage (CD86+ and CD206+) infiltrations were assessed. *In vitro*, peritoneal macrophages (PM_Φ_) were isolated from wild-type and β_2_AR-knockout female mice. The PM_Φ_ were treated with ISO, E_2_, and β_2_AR blocker ICI 118,551 for 24 h, and flow cytometric evaluations were done to assess their phenotypic expression. E_2_ deficiency permitted the induction of CSC, which was characterized by cardiac dysfunctions, maladaptive myocardial hypertrophy, unresolved proinflammatory responses, and fibrosis. Nonetheless, E_2_ presence/supplementation during stress averted all the aforementioned adverse effects of chronic stress while preventing excessive depletion of β_2_AR. Also, we demonstrated that E_2_ facilitates timely resolution of myocardial proinflammation to permit reparative functions by enhancing the polarization of CD86+ to CD206+ macrophages. However, this adaptive immunomodulation is hampered when β_2_AR is inhibited. Taken together, the outcomes of this study show that E_2_ confers cardioprotection to prevent CSC *via* adaptive immunomodulation of macrophage phenotypes, and β_2_AR-mediated signaling is crucial for the polarizations of CD86+ to CD206+ macrophages.

## Introduction

Similar to other cardiovascular diseases (CVDs), chronic stress-induced cardiomyopathy (CSC) has been clinically shown to be predominant in males of all age cohorts, while females are mostly predisposed to its occurrence during their menopausal period (Boese et al., 2017; Ndzie Noah et al., 2021). Recent attempts to elucidate the underlying disease mechanisms have revealed crucial roles played by estrogen during stress to sustain good cardiac health in premenopausal women, besides its reproductive functions (Mori et al., 2011; Boese et al., 2017).

Typically, the clinical manifestations of CSC patients are severe left ventricular (LV) diastolic dysfunction (LVDD) and systolic dysfunctions (LVSD) (Medeiros et al., 2014). The adverse structural remodeling includes excessive LV hypertrophy and massive interstitial fibrosis, which results in the stiffening of the myocardia and causes these cardiac dysfunctions. Also, recent studies have demonstrated that unresolved myocardial inflammatory responses characterized by the enormous influx of proinflammatory macrophages exacerbates CSC and aggravates adverse cardiac remodeling (Wilson et al., 2018; Scally et al., 2019).

Under physiological state, inotropic and chronotropic functions of the heart are mediated by β-adrenergic receptors (βARs) *via* G stimulatory protein (G_*s*_), mainly β_1_AR and β_2_AR. However, hyperstimulation of the receptors during chronic stress results in the downregulation of β_1_AR mostly, while β_2_AR traffics the stimuli signal *via* G inhibitory protein (G_*i*_) to prevent cardiac insults (Paur et al., 2012). The homologous desensitization of βARs which results in the downregulation is induced by G protein-coupled receptor kinases 2 (GRK2) phosphorylation and β-arrestin-1 recruitment (Adzika et al., 2019). Nonetheless, it was demonstrated in previous studies that estrogen ameliorates stress-induced cardiac insults by preventing excessive depletion of β_2_ARs and also facilitating a balance in the G_*i*_/G_*s*_ signaling pathways (Cao et al., 2015; Hou et al., 2018). The estrogenic effects of sustaining β_2_AR activities during stress might be likely due to its inhibitory effects on GRK2 (Abraham et al., 2018). Intriguingly, β_2_AR are profoundly expressed on macrophages; hence, estrogen may indirectly modulate their activities and possibly their polarizations into proinflammatory (CD86+ macrophages) or anti-inflammatory (CD206+ macrophages) phenotypes in the myocardia. It is hypothesized that the possible exertion of the aforementioned estrogenic immunoregulation might prevent extensive pathological cardiac remodeling during chronic stress by subduing maladaptive myocardial hypertrophy, fibrosis, and proinflammatory responses.

Herein, we sought to explore the cardioprotective mechanisms employed by estrogen *via* immunomodulation to decrease the incidence of CSC in female rat models, as understanding these mechanisms will provide the basis for further translational research into preventing CSC in postmenopausal women.

## Materials and Methods

### Experimental Animals and Models

The wild-type and β_2_AR knockout FVB female mice (donated by Professor Daniel Bernstein, Stanford University—United States) and adult female Sprague-Dawley (SD) rats (180–200 g) were used for the experiments (*n* = 4–8 rats/group). All standard animal house boundary protocols were observed. After ensuring the SD rats were in the same menstrual phase through vaginal mucus examination, bilateral ovariectomy (OVX) and sham surgeries were done as we previously described (Zhang et al., 2021).

**FIGURE 1:**
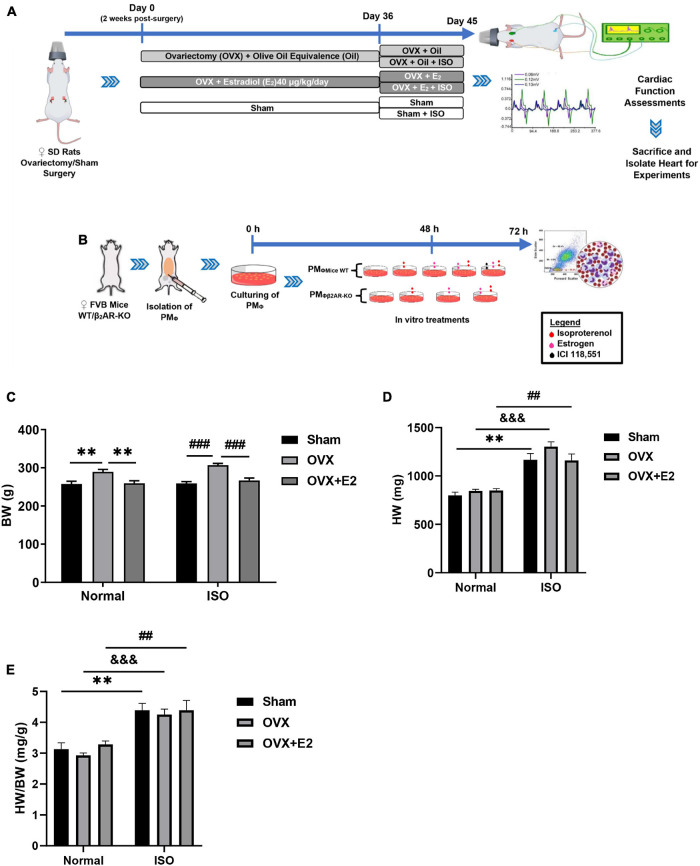
**(A,B)** Illustration of the experiment timeline for making *in vivo* and *in vitro* models, respectively. **(C–E)** Graphical presentations of morphometric data demonstrate alterations in body weight (BW), heart weight (HW), and HW/BW coefficient during chronic stress and estrogen deficiency (*n* = 8 rats/group). **p < 0.01; ^##^*p* < 0.01 and ^###^*p* < 0.001; ^$$$^*p* < 0.001. Data are presented as mean ± SEM. Data were analyzed using two-way ANOVA, followed by Sidak’s *post hoc* analysis.

As illustrated (Figure 1A), 2 weeks after ovariectomy, the rats were intraperitoneally injected with 40 μg/kg/day of estradiol (E_2_) (E2758; Sigma, St. Louis, MO, United States) for 31 days, as done previously (Zhang et al., 2021). Olive oils of equivalent amounts were administered as a placebo to the control groups. On day 36 after ovariectomy, chronic stress was induced in rats that were being treated with either E_2_ or the placebo by subcutaneous injections of isoproterenol (ISO) (160504; Sigma) at 5 mg/kg/day for 10 days, as previously done (Lin et al., 2016; Zhou et al., 2017). Also, the Sham surgery rats had similar ISO and placebo treatments. In total, *in vivo* experiments included the following six groups; (i) Sham group, (ii) OVX group, (iii) OVX + E_2_ group, (iv) Sham + ISO group, (v) OVX + ISO group, and (vi) OVX + ISO + E_2_ group.

The dosage of E_2_ employed was to mimic its physiological levels in rats, as we had demonstrated previously (Liu et al., 2012; Zhang et al., 2021). Also, rather than the high dosage of ISO used in acute stress models, as done previously (Youssef et al., 2021), a relatively milder dosage was used due to the prolonged duration (10 days) of the catecholamine treatment (Zhang et al., 2021).

### Hemodynamic Experiment

After properly sedating rats (*n* = 7 rats/group), they were fixed in the supine position, and longitudinal incisions (about 2 cm in length) were made in the mid-neck. By using blunt hemostatic forceps, the fascia and aponeurosis were separated to reveal 1–1.5 cm of the right common carotid artery. The distal end of the right common carotid artery was ligated, and the proximal end of the right common carotid artery was clamped with an arterial clamp. Next, an ophthalmic scissor was used to nick the artery (in a V-shaped), a heparin-filled catheter attached to a pressure transducer was carefully inserted into the left ventricle. The left ventricular systolic and end-diastolic pressures and electrocardiography (ECG) were recorded with PowerLab data acquisition system (ADInstruments, North America, COlorado Springs, CO, United States).

### Histological Assessment of Myocardia

Excised hearts (*n* = 6 hearts/group) were properly washed with prechilled PBS, blotted with filter paper, and fixed in 4% paraformaldehyde for more than 48 h. Next, the heart specimens were embedded in paraffin, sectioned at 4 μm thickness, and preserved for histological assessments.

The myocardial sectionings were deparaffinized before performing Masson’s trichrome (Maxim Biotechnologies, Fuzhou, China), hematoxylin and eosin (H&E), and immunohistochemical (IHC) staining as previously described (Zhang et al., 2021). The trichome staining were done to ascertain the collagen volume fraction (CVF) while H&E staining were done to assess cardiomyocyte diameters and help depict the extent of myocardial hypertrophy. Also, IHC staining with CD68 (Abcam, Cambridge, United Kingdom; ab955), CD86 (Bioss, Woburn, MA, United States; BS-1035R), and CD206 (Abcam; ab8918) was done to assess the extent of myocardial infiltrations of inflammatory cells.

Imaging of all stained sections were done at × 400 magnification (IX 71, Olympus, Tokyo, Japan) and analyzed using ImageJ (1.53a version; National Institute of Health, Bethesda, MD, United States).

### Quantitative Real-Time PCR

Trizol (Invitrogen Co., Carlsbad, CA, United States) was used to extract RNAs from homogenized myocardia (*n* = 4 hearts/group). After the normalization of RNA concentrations, cDNAs were synthesized using Revertra ace^®^ qPCR rt kit (Toyobo, Osaka, Japan). By using SYBR Green Master Mix (Vazyme Biotech, Nanjing, China), the following gene primers (Sangon Biotech, Shanghai, China) were used to evaluate mRNA expressions; (1) Tumor necrosis factor-alpha (TNF-α), Forward GAAAGCATGATCCGAGATGTG; Reverse: CACGAGCAGGAATGAGAAGAG, (2) transforming growth factor-beta (TGF-β1), Forward: ATGGTGGACCGCA ACAACGC; Reverse: CTGGCACTGCTTCCCGAATGTC, (3) inducible nitric oxide synthase (iNOS), Forward: TCTTGGAGCGAGTTGTGGATTGT; Reverse: TAGGTGAGG GCTTGCCTGAGTG, (4) arginase 1 (Arg-1), Forward: CGTTG ACCTTGTCTTGTTTTGG; Reverse: CTGGTTCTGTTCGGT TTGCTG, (5) glyceraldehyde 3-phosphate dehydrogenase (GAPDH), Forward: TCCTGCACCACCAACTGCTTAG; Reverse: AGTGGCAGTGATGGCATGGACT.

The 2^–ΔΔ*Ct*^ analysis method was used to evaluate the relative mRNA levels as described (Gold et al., 2012) and have been graphically presented as fold changes compared with the Sham group.

### Western Blotting

Proteins were extracted from myocardial apexes (*n* = 4 hearts/group), treated with reducing agents, denatured at 100°C, and separated by gel electrophoresis as previously described (Hou et al., 2018). Next, the proteins were transferred onto polyvinylidene fluoride (PVDF) membranes, blocked with 1% bovine serum albumin, and incubated in the following primary antibodies at 4°C overnight; ANP (1:1,000, Santa Cruz Biotechnology, Dallas, TX, United States; sc-515701), BNP (1:1,000, Santa Cruz Biotechnology; sc-271185), β_2_AR (1:1,000, Abcam; ab182136), GAPDH (1:4,000, Proteintech, Manchester, United Kingdom; 10494-1-AP). Visualizations of immunoblots were done with enhanced chemiluminescence (Tanon, Shanghai, China). The protein bands were quantified and evaluated by the relative expressions with their GAPDH.

### Isolation and Characterization of Peritoneal Macrophages for *in vitro* Experiments

Peritoneal macrophages (PM_Φ_) (*n* ≤ 2 * 10^6^ cells) were harvested from wild-type (WT) and β_2_AR knockout (β_2_AR-KO) FVB female mice by using methods previously demonstrated (Ray and Dittel, 2010). In brief, the mice peritoneum were exposed under aseptic conditions. Five to 10 ml of prewarmed (37°C) 3% fetal bovine serum (FBS) were injected into the peritoneal cavity. Cell suspensions were collected after softly massaging for 5 min and centrifuged at 1,500 rpm for 10 min, and the obtained cell pellets were resuspended and cultured with 10% FBS at 37°C and 5% CO_2_ for 48 h. Next, 24 h *in vitro* treatments of cultured PM_Φ_ included; ISO (10 μM), E2 (1 nM), and β_2_AR blocker ICI 118,551 (55 nM) (Figure 1B). These treatments were preceded by E2 pretreatments for 1 h, in groups where the estrogenic effects were to be ascertained.

The identification and subtyping of isolated PM_Φ_ after culturing or treatments were done by flow cytometry BD LSR II (BD Biosciences, San Jose, CA, United States). APC anti-F4/80 (123116; BioLegend, San Diego, CA, United States) and FITC anti-CD11b (101206; BioLegend) antibodies were used to identify the macrophages while PerCP anti-CD86 (105028; BioLegend) and PE anti-CD206 (141706; BioLegend) antibodies were used to differentially identify M1 macrophages and M2 macrophages, respectively. Preparations of cultured or treated PM_Φ_ for flow cytometry were done as previously described (Zhu et al., 2017). Acquired data were analyzed with FlowJo software (v10; FlowJo LLC, Oregon, OR, United States).

### Statistical Analysis

Statistical analysis was performed with GraphPad Prism 5.0 (GraphPad Software, San Diego, CA, United States). All data were presented as mean ± SEM and compared by two-way ANOVA. *p-*values < 0.05 were deemed statistically significant.

## Results

### Estrogen Deficiency Facilitates Weight Gains During Chronic Stress

Analysis of the morphometrics of rats demonstrated that E_2_-deficient (OVX) rats gained significant body weights (BW). This phenomenon is shown to have been further aggravated by chronic stress (ISO) and is accompanied by increases in heart weights (HW) (Figures 1C,D). However, the supplementation with exogenous E_2_ (E_2E__*xo*_) in the OVX + E_2_ group and endogenous E_2_ (E_2E__*ndo*_) in the Sham group helped to significantly prevent BW gains and slight decrease HW (without statistical significance on comparing among the stress groups). Furthermore, it is shown that the HW/BW coefficient variation between physiological and stress states is more significant in OVX rats (Figure 1E).

### Estrogen Deficiency Aggravates Isoproterenol-Induced Cardiac Dysfunction

**FIGURE 2:**
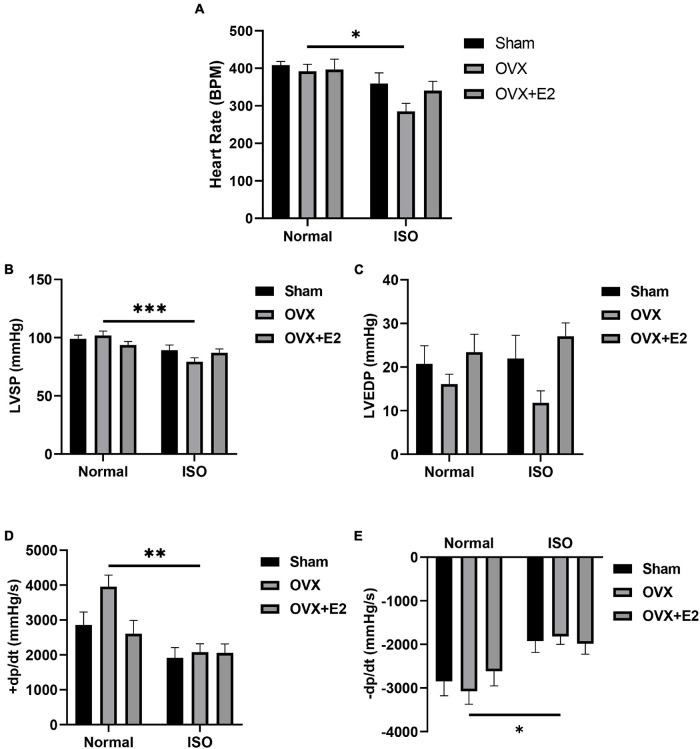
Estrogen deficiency permits cardiac dysfunction chronic stress. **(A)** Graphical representation of heart rates (HR). **(B,C)** Left ventricular systolic pressure (LVSP) and left ventricular end-diastolic pressure (LVEDP) recordings depict cardiac dysfunction in OVX + ISO rats. **(D,E)** Rate of pressure development (+ dp/dt) and the rate of pressure development decay (−dp/dt) further validate cardiac dysfunction in OVX + ISO rats. (*n* = 7 rats/group). *p < 0.05, **p < 0.01, and ***p < 0.001. Data are presented as mean ± SEM. Data were analyzed using two-way ANOVA and Bonferroni’s multiple comparisons test.

E_2_ deficiency during chronic stress resulted in decreased heart rates (HR) in OVX rats. The supplementation of E_2E__*xo*_ in OVX rats and the presence of E_2E__*ndo*_ in Sham rats prevented a significant decrease in HR during chronic stress (Figure 2A). Furthermore, the cardiac function index; LVSP, LVEDP, the rate of pressure development (+dp/dt), and the rate of pressure development decay (−dp/dt) were assessed to ascertain for any occurring dysfunctionalities. It was demonstrated that E2 deficiency during chronic stress resulted in depressions in LVSP, LVEDP, +dp/dt, and −dp/dt. However, E_2E__*ndo*_ and E_2E__*xo*_ prevented significant alterations in these cardiac function indexes during stress (Figures 2B–E).

### Estrogen Deficiency Promotes Myocardial Hypertrophy and Fibrosis During Chronic Stress

**FIGURE 3:**
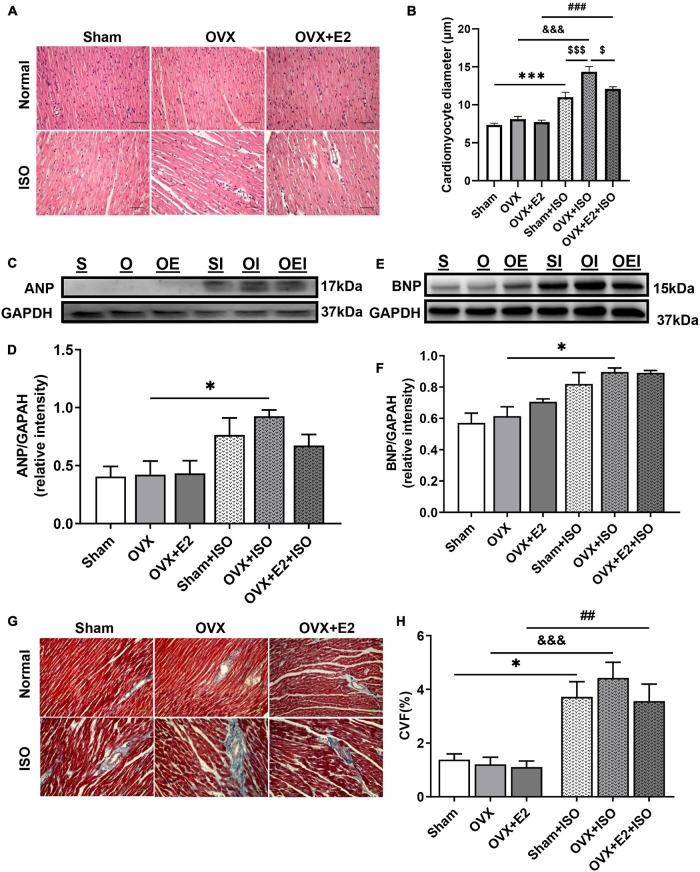
Estrogen deficiency promotes myocardial hypertrophy and fibrosis during chronic stress. **(A,B)** Representative H&E staining and graphical presentation of measured cardiomyocyte diameters, respectively (*n* = 10–12 cells/5 field of view/6–8 sections/6 hearts/group). **(C–F)** Representative immunoblots and graphical presentations of assessed cardiac hypertrophy markers, atrial natriuretic peptide (ANP), and brain natriuretic peptide (BNP) (*n* = 4 hearts/group). **(G,H)** Representative Masson’s trichrome staining and graphical presentation of evaluated collagen volume fractions to assess the extent of fibrosis (*n* = 5–7 field of view/6–8 sections/6 hearts/group). *p < 0.05 and ***p < 0.001; ^##^*p* < 0.01 and ^###^*p* < 0.001; ^$^*p* < 0.05 and ^$$$^*p* < 0.001; ^&&&^*p* < 0.001. Data are presented as mean ± SEM. Data were analyzed using two-way ANOVA and Bonferroni’s multiple comparisons test.

To ascertain the impact of chronic stress on the myocardial architecture, H&E and trichrome staining were done to evaluate the extent of cardiomyocyte hypertrophy and interstitial collagen deposition, respectively. The measurements of cardiomyocyte diameters from H&E-stained myocardia across all groups demonstrated that, under physiological state, the deficiency of E_2_ does not affect the cell sizes. However, E_2_ deficiency (in OVX rats) during chronic stress permits excessive cardiomyocyte hypertrophy. Also, the obtained results showed that, while E_2_ in general inhibited excessive cardiomyocyte hypertrophy during stress in both Sham and OVX + E_2_ groups, E_2E__*ndo*_ (in Sham) exhibited much more potent antihypertrophic effects than E_2E__*xo*_ (in OVX + E_2_) did (Figures 3A,B). Next, immunoblotting of atrial natriuretic peptide (ANP) and brain natriuretic peptide (BNP) depicted the maladaptive nature of the resulting cardiomyocyte hypertrophy when E_2_ is deficient during stress. E_2E__*ndo*_ relatively decreased the expressions of both natriuretic peptides; whereas, E_2E__*xo*_ only affected ANP upregulations (Figures 3C–F).

Assessed CVF demonstrated that myocardial interstitial fibrosis increases during chronic stress in OVX rats; however, the results trend showed that the presence of E_2_ does ameliorate its severity but without statistical significance on comparing with OVX + E2 + ISO and Sham + ISO groups (Figures 3G,H).

### Estrogen Attenuates Maladaptive Myocardial Inflammatory Responses During Chronic Stress

**FIGURE 4:**
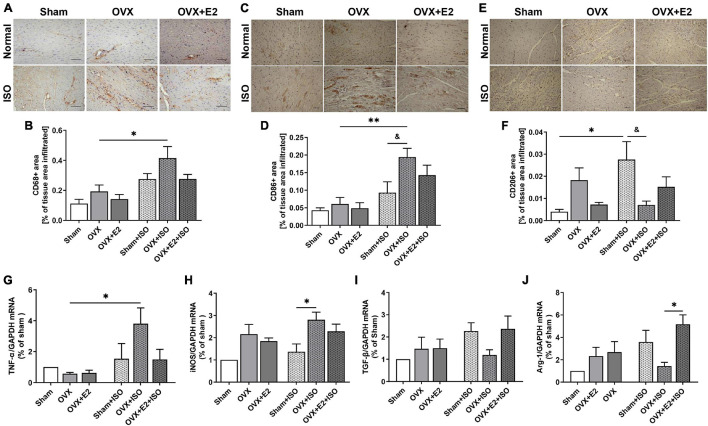
Estrogen attenuates maladaptive myocardial inflammatory responses chronic stress. **(A,B)** Representative immunohistochemical staining and graphical presentation of CD68-positive cells (whole macrophages) assessed from the myocardia. **(C,D)** Representative immunohistochemical staining and graphical presentation of CD86-positive cells (proinflammatory phenotype/M1 macrophages) assessed from the myocardia. **(E,F)** Representative immunohistochemical staining and graphical presentation of CD206-positive cells (anti-inflammatory phenotype/M2 macrophages) assessed from the myocardia (*n* = 6–8 field of view/6–8 sections/6 hearts/group). **(G,H)** Graphical presentation of M1 macrophage markers, tumor necrosis factor-alpha (TNF-α), and inducible nitric oxide synthase (iNOS) mRNA expressions assessed by RT-qPCR (*n* = 4 hearts/group). **(I,J)** Graphical presentation of M2 macrophage markers, transforming growth factor-beta (TGF-β1), and arginase 1 (Arg-1) mRNA expressions assessed by RT-qPCR (*n* = 5 hearts). *p < 0.05 and **p < 0.01; ^&^*p* < 0.05. Data are presented as mean ± SEM. Data were analyzed using two-way ANOVA and Bonferroni’s multiple comparisons test.

Myocardial inflammation during chronic stress contributes to aggravated cardiac remodeling (Hulsmans et al., 2018). Hence, we assessed the potentials of E_2_ in exerting adaptive immunoregulation in the myocardia during stress. CD68-positive IHC staining demonstrated that, under physiological state, the amount of macrophages infiltrating the myocardia are slightly elevated when E_2_ is deficient (in OVX rats). Also, although CD68-positive cell infiltrations were generally increased during chronic stress, significant upregulations only resulted in OVX + ISO rats. The presence of E_2E__*ndo*_ and E_2E__*xo*_ in Sham + ISO and OVX + E_2_ + ISO, respectively, prevented enormous CD68-positive cell infiltration into the myocardia during stress (Figures 4A,B). Furthermore, by using CD86 and CD206 IHC staining, it is shown that majority of the inflammatory cells infiltrating the myocardia during stress when E_2_ is deficient are CD86-positive (proinflammatory) cells, while CD206-positive (anti-inflammatory) cell infiltrations are significantly hampered. However, the contrast of this phenomenon is demonstrated by E_2E__*ndo*_ and E_2E__*xo*_ presence in Sham + ISO and OVX + E_2_ + ISO, respectively, during stress. The anti-inflammatory cell infiltrations are significantly increased while proinflammatory cell infiltrations were dampened in these groups. Also, it is observed that E_2E__*ndo*_ was potent than E_2E__*xo*_ in the adaptive modulation of myocardial inflammatory cell infiltrations (Figures 4C–F). To validate the adaptive immunoregulation exerted by E_2_, mRNAs of proinflammatory (TNF-α and iNOS) and anti-inflammatory (TGF-β1 and Arg-1) biomarkers were assessed from the myocardia. During chronic stress, E_2_ deficiency (in OVX rats) permitted upregulations of TNF-α and iNOS while TGF-β1 and Arg-1 expressions were downregulated. Conversely, E_2_ enhanced the expressions of TGF-β1 and Arg-1 and decreased TNF-α and iNOS levels (Figures 4G–J).

### Estrogenic Adaptive Immunoregulation of Macrophage Polarization Involves Modulation of β_2_AR Signaling Activities

**FIGURE 5:**
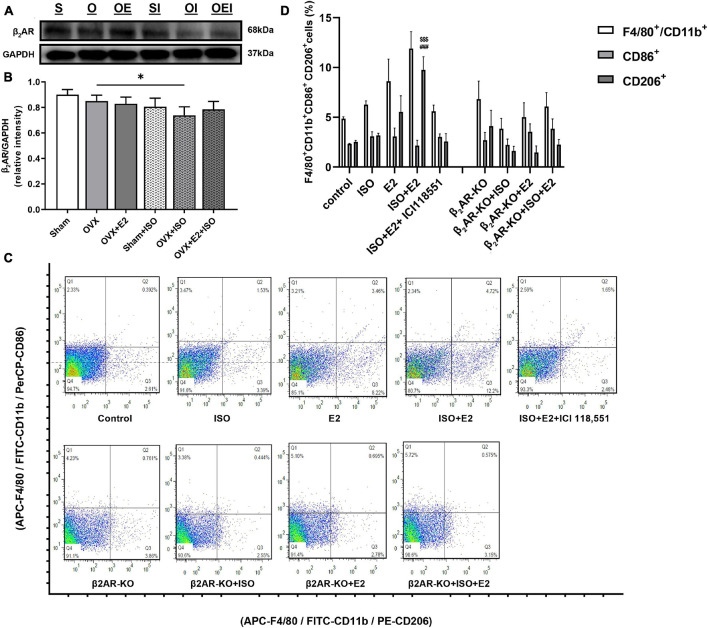
Estrogenic adaptive immunoregulation of macrophage polarization involves modulation of β_2_AR signaling activities. **(A,B)** Representative immunoblots and graphical presentation of β_2_AR expressions evaluated from the myocardia. S, Sham; O, OVX; OE, OVX + E_2_; SI, Sham + ISO; OI, OVX + ISO; OEI, OVX + E_2_ + ISO (*n* = 4 hearts/group). **(C,D)** Representative flow cytometry plots of gated macrophage phenotypes and graphical presentation of their M1 and M2 expression ratios (*n* ≤ 1 * 10^6^ cells). The phenotypic populations of macrophages were quantified using FlowJo. *p < 0.05; ^$$$^*p* < 0.001 vs. CD86+ (ISO + E2); ^###^*p* < 0.001 vs. CD206+ (ISO + E2 + ICI118551). Data are presented as mean ± SEM. Data were analyzed using two-way ANOVA, followed by Sidak’s *post hoc* analysis.

In our previous studies (Hou et al., 2018), it was demonstrated that E_2_ conferred cardioprotective effects *via* the modulation of β_2_AR-G_α*s*_/G_α*i*_-mediated signaling cascades during stress. Hence, to elucidate the underlying mechanism employed by E_2_ to facilitate timely resolutions of myocardial proinflammatory responses, we again investigated the likely involvement of β_2_AR since they are well expressed in both cardiomyocytes and macrophages. Immunoblotting results showed a significant decrease in β_2_AR expression in OVX + ISO rats (Figures 5A,B). However, the extent of β_2_AR downregulations in Sham + ISO and OVX + E_2_ + ISO was relatively lower than OVX + ISO, which showed statistical significance when compared with OVX. Flowcytometry evaluations of PM_Φ_ isolated from WT and β_2_AR-KO and treated with ISO (10 μM) and/or E_2_ (1 nM) along with or without β_2_AR blocker ICI 118,551 (55 nM), demonstrated that the inhibition or obliteration of β_2_AR abolished the adaptive immunoregulatory effects exerted by E_2_ during chronic stress. Typically, it is shown that during stress, E_2_ enhanced PM_Φ_ polarizations into more CD206+ macrophages (anti-inflammatory phenotype) than CD86+ macrophages (proinflammatory phenotype) when β_2_ARs are not inhibited. However, obliteration of β_2_AR activities (by its KO or blocker ICI 118,551) obstructs the initially observed estrogenic phenomenon and consequently causes an increase in M1 macrophage phenotype (Figures 5C,D).

## Discussion

Unresolved myocardial inflammatory responses have been clinically demonstrated as an underlying factor expediting the pathological remodeling of the heart during stress (Mori et al., 2011; Wilson et al., 2018; Scally et al., 2019). The homeostatic balance between cardiac proinflammatory and anti-inflammatory macrophage phenotypes is crucial for resolving myocardial inflammation and proper heart functioning (Mouton et al., 2018). However, clinical studies have shown that the myocardia of CSC patients have massive bias infiltrations of proinflammatory macrophages, which prolongs inflammation without timely resolutions to permit reparative functions of anti-inflammatory macrophages. Hence, in post-stress–induced cardiac injuries, the maladaptive proinflammatory responses in the myocardia drives the pathological remodeling of the heart, which is evident by marked fibrosis (Mori et al., 2011; Mouton et al., 2018).

Herein, we demonstrate the mechanistic roles employed by E_2_ to protect the heart during chronic stress from an immunoregulatory perspective. Morphometric evaluations revealed significant gains in BW resulting from the deficiency of estrogen in the OVX rats under physiological and chronic stress states. This finding provides supporting evidence that E_2_ is crucial for efficient lipid metabolism. In fact, previous studies have demonstrated that E_2_ maintains a healthy lipid profile by upregulating bloodstream levels of high-density lipoprotein (HDL) and lowering low-density lipoprotein receptors (LDL) (Lee et al., 2015; Fu et al., 2019; Ndzie Noah et al., 2021). As such, the deficiency of E_2_ scaffolded disorders in lipid metabolism that caused weight gain as it permitted increased circulation LDL (bad cholesterol) level which deposited as adipose all over the body as well as in and around vascular tissues and circulatory organs (Lee et al., 2015; Kozakowski et al., 2017). Similar to the previous work of Ren et al. (2003), BW was increased in OVX rats. Also, it was observed that the combination of E_2_ deficiency and stress increased HW and HW/BW coefficient more in OVX rats. The HW and HW/BW coefficient increases may be due to increased epicardial adipose and cardiomyocyte hypertrophy. Intriguingly, epicardial adipose has been shown to be a reservoir for macrophages which infiltrates the myocardia to hasten maladaptive inflammatory responses (Mori et al., 2011). Therefore, the increased accumulation of epicardial adipose resulting from E_2_ deficiency predisposes the heart to sustained myocardial inflammation should there be any cardiac insult during stress. Overall, consistent with early findings (Ren et al., 2003; Mori et al., 2011; Michalson et al., 2018), it was demonstrated that E_2E__*ndo*_ and its supplementation (E_2E__*xo*_) prevents excessive weight gains, which ultimately impacts positively on cardiac health.

Clinically, demographics clearly show that normally, females have higher heart rates (HR) and cardiac outputs than males of the same age cohort (Wheatley et al., 2014). In menopause, there is a further increase in HR, which results in short-term arrhythmias (heart palpitations) and are attributed to the loss of E_2_ and possibly β_2_AR signaling dysregulation (Carpenter et al., 2021). Interestingly, the contrary was found in this study. The obliteration of E_2_ *via* ovariectomy resulted in a slight decrease in HR under normal state; however, chronic stress in these OVX rats caused a significant reduction in HR. The possible explanation for this outcome is that inotropy and chronotropic functions of the heart are mediated by β_1_AR and β_2_AR; meanwhile, E_2_ prevents their dysregulations and substantial depletion during stress (Hou et al., 2018; Ndzie Noah et al., 2021). Therefore, E_2_ deficiency might have permitted dysfunctionalities and downregulation of the β_2_ARs during stress, hence the significant decrease in HR. Also, consistent with previous reports, it was found that the cardiac function index; LVSP, LVEDP, +dp/dt, and −dp/dt were unaffected by E_2_ deficiency under physiological state (Mori et al., 2011; Ribeiro et al., 2013). Even so, chronic stimulation of βARs by ISO during E_2_ deficiency demonstrated overt cardiac dysfunctionalities. In contrast, it was demonstrated that E_2E__*ndo*_ and E_2E__*xo*_ in the Sham + ISO and OVX + E2 + ISO rats, respectively, ameliorated these heart dysfunctions to sustain cardiac output during stress.

Further investigations sought to characterize the impact of chronic stress on the myocardial structure during E_2_ deficiency. It was observed that cardiomyocyte diameters generally increased during stress; however, the E_2_ deficiency permitted maladaptive hypertrophy, which distorted the typical myocardial architecture. This was further proven by the significant upregulations of ANP and BNP in the hearts of OVX rats during chronic stress. Nevertheless, E_2E__*ndo*_ (in the Sham rats) showed much potency at minimizing the upregulations of both ANP and BNP during stress, while E_2E__*xo*_ (in the OVX + E_2_ rats) was unable to downregulate the latter substantially. In conformity with our findings, Goncalves et al. (2018) and others had early demonstrated that E_2_ exerts antihypertrophic effects *via* GPER (Goldstein et al., 2004). Also, the discrepancies observed between the antihypertrophic effect of E_2E__*ndo*_ and E_2E__*xo*_ might have occurred because other ovarian secretions such as vascular endothelial growth factor (VEGF) may complement the efforts of E_2_ in preventing maladaptive cardiomyocyte hypertrophy (Cai et al., 2015). Besides, as suggested by Zhang et al. (2021), unlike the E_2E__*xo*_ treatment dose, which remained constant during CSC modeling, the levels of E_2E__*ndo*_ are altered due to the estrous cycle in the Sham and could have also contributed to the observed differences in the antihypertrophic effect of E_2_. In addition, it was found that obliteration of E_2_ in OVX rats permitted induction of massive interstitial fibrosis; nevertheless, its presence/restoration ameliorated this adverse outcome. We showed that comparatively, E_2E__*ndo*_ in the Sham and its supplementation (E_2E__*xo*_) lessened the extent of fibrosis, just as demonstrated earlier (Mori et al., 2011; Michalson et al., 2018).

The homeostatic balance between proinflammatory and anti-inflammatory macrophages in the myocardia during steady state is crucial for cardiac function, as is the timely trafficking of either of them during injury/cell clearance or reparative process, respectively, essential for preventing adverse heart remodeling (Lavine et al., 2014; Mouton et al., 2018). However, as demonstrated from the postmortem examination of the hearts from CSC patients, proinflammatory macrophages were abundant in the myocardia and were shown to have exacerbated myocardial proinflammatory responses, which may have resulted from stress-induced cardiac insults. The observed biased infiltration of CD86+ macrophages (proinflammatory) hastened the pathological cardiac remodeling as autopsied hearts had marked fibrosis (Wilson et al., 2018; Scally et al., 2019). Similar to these clinical findings, it has been shown in rats that stress causes augmentation of myocardial CD86+ macrophage infiltrations, and the phenomena are worsened by E_2_ deficiency (Mori et al., 2011). Following up on these previous studies, consistent findings were made. CD68-positive cell infiltration into the myocardia were increased only under stress conditions; however, E_2_ deficiency augmented their infiltration significantly. Nonetheless, E_2E__*ndo*_ and its supplementation (E_2E__*xo*_) to the rats during stress minimized CD68-positive cell infiltration. Assessing the phenotypic ratios with CD86 and CD206 immunostaining revealed the majority of the CD68-positive cells infiltrating the myocardia when E_2_ is deficient during stress are CD86-positive cells, while CD206-positive cells are less present. Nevertheless, E_2E__*ndo*_ and E_2E__*xo*_ reversed these phenomena by enhancing anti-inflammatory responses in the hearts during stress *via* increasing CD206+ macrophage presence, as similarly reported previously (Xing et al., 2009; Bolego et al., 2013). Validations of the aforementioned findings were done by assessing the mRNA expressions of proinflammatory (TNF-α and iNOS) and anti-inflammatory macrophage (TGF-β and Arg-1) markers from the myocardia of all experimental groups. Similar to the observations of the histological evaluations, TNF-α and iNOS were upregulated during stress and were further elevated significantly when E_2_ is deficient. Also, TGF-β and Arg-1 mRNA expressions were downregulated in the myocardia due to E_2_ deficiency. Conversely, E_2E__*ndo*_ exerted anti-inflammatory effects by enhancing TGF-β and Arg-1 while decreasing TNF-α and iNOS mRNA expressions during stress. Although E_2E__*xo*_ upregulated TGF-β and Arg-1 and inhibited TNF-α similarly to E_2E__*ndo*_, it was not as potent as E_2E__*ndo*_ in downregulating iNOS. The possible explanation of the phenomenon is that ovarian secretions of progesterone might have complimented the inhibitory effects of E_2E__*ndo*_, as it has been reported that besides E2, progesterone decreases iNOS levels in non-cardiac tissue (Menzies et al., 2011). However, progesterone is obliterated in OVX + E2 + ISO rats; hence, it might account for iNOS being significantly downregulated in Sham + ISO than OVX + E2 + ISO. Nonetheless, the estrogenic anti-inflammatory effects demonstrated here have been similarly reported by Villa et al. (2015) and others (Chen et al., 2021).

Similar to cardiomyocytes, macrophages have profound expressions of β_2_AR and estrogen receptors (ERs), and the cardioprotective effects conferred by E_2_ have been demonstrated to mostly involved the synergy of ERs and β_2_AR signaling cascades (Kang et al., 2012; Hou et al., 2018; Machuki et al., 2019; Ndzie Noah et al., 2021). Hence, to elucidate the immunoregulatory mechanisms employed by E_2_ to facilitate more CD206+ macrophage polarizations to accelerate the resolution of myocardial inflammation during stress, we investigated the possible involvement of β_2_AR signaling modulation by E_2_-induced cascades. In conformity with our initial speculations, β_2_AR expressions from apical myocardia (constituting cardiomyocytes and infiltrated macrophages) were found to be significantly depleted during chronic stress due to E_2_ deficiency, as the presence of E_2E__*ndo*_ in the Sham and the supplementation of E_2E__*xo*_ in OVX rats showed a minimal reduction in the expression of the receptor under the same stress condition. Further investigations of β_2_AR involvement deployed the isolations of PM_Φ_ from female WT and β_2_AR-KO mice as well as the use of β_2_AR blocker ICI 118,551 to ascertain if E_2_ induced any variations in the phenotypic ratios of the macrophages during stress was affected by impeding β_2_AR signaling. We report that the estrogenic signaling facilitates adaptive immunoregulation by ensuring CD206+ macrophage polarizations to timely resolve inflammation as reported by others (Keselman et al., 2017). However, for the first time, we show the underlying mechanism involves interplays of E_2_, ERs and β_2_AR signaling during stress. Flow cytometric evaluations show that E_2_ treatments during stress increased CD206+ macrophage polarizations against CD86+ macrophages; however, the deletion/inhibition of β_2_AR impaired this phenomenon. These observations are possibly because the bioavailability of nitric oxide (NO), which is produced *via* β_2_AR-G_*ai*_-PI3K-Akt–mediated signaling cascade, is crucial for the polarization of macrophages from proinflammatory to the anti-inflammatory phenotype (De Nigris and Prattichizzo, 2021). As such, blockade of β_2_AR signaling disrupts NO bioavailability and abolishes this adaptive immunoregulatory mechanism. Also, E_2_ had been shown previously to exert these anti-inflammatory effects primarily *via* estrogen receptor alpha (ERα) (Bolego et al., 2013; Campbell et al., 2014), and although we have demonstrated here the essential involvement of β_2_AR to facilitate the polarizations of CD206+ macrophages, there are apparent interplays among E_2_, ERs, and β_2_AR to ensure this immunomodulation during stress. Intriguingly, E_2_ and ER activities downregulate GRK2, which otherwise would have induced the homologous desensitization and downregulation of β_2_AR during stress (Abraham et al., 2018; Arcones et al., 2021). Therefore, E_2_ and ERs indirectly sustain the bioavailability of NO *via* β_2_AR-G_*ai*_-PI3K-Akt signaling by preventing dysregulation of the receptor during stress and enhancing the β_2_AR-mediated CD206+ macrophage polarization.

Taken together, the findings from this study demonstrate the immunoregulatory mechanisms employed by E_2_ to confer cardioprotection and lower the incidence of CSC in premenopausal women as compared with postmenopausal women and males of all age cohorts. E_2_ exerts this immunoregulatory myocardia protection to prevent pathological cardiac remodeling during stress by ensuring the timely resolution of myocardial proinflammatory responses and enhancing reparative functions of CD206+ macrophage. More importantly, we demonstrate here that the adaptive modulation of macrophage phenotypes by E_2_ during stress requires the mediation of β_2_AR signaling. The classical interplays among E_2_, ERs, and β_2_AR discussed by Ndzie Noah et al. (2021) are also shown here, as E_2_ and ER activities are in turn required to prevent β_2_AR dysregulations and dysfunctionalities during stress. From a therapeutic standpoint, the findings from this study reechoes the essence of E_2_ replacement therapy (E_2_RT) in postmenopausal women, as it reduces the incidence of CSC. However, it is recommended that E_2_RT is initiated within 5–6 years after menopause so as to explore its therapeutic benefits fully while circumventing the adverse outcomes reported by the Women’s Health Initiative from their randomized controlled trial (Rossouw et al., 2002; Michalson et al., 2018; Ndzie Noah et al., 2021). Finally, it is deemed necessary to point out the limitations of this study due to its clinical significance. β_1_ARs are essential for myocardial functions and might play other immunologic roles facilitated by E_2_, but they have not been elucidated previously nor in this study. Also, in some instances (Figures 3E,F), (Figures 4C–J), it is shown that E_2E__*xo*_ did not confer anti-inflammatory effects as E_2E__*ndo*_ did. However, the fact that other ovarian secretions such as progesterone can complement the anti-inflammatory effects of E_2E__*ndo*_ but are obliterated by ovariectomy in the E_2E__*xo*_ treatment group might explain the observed differences. The estrous cycle in the Shams causing alterations in E_2E__*ndo*_ levels while E_2E__*xo*_ treatment dosage used remained constant might also account for the shown slight variations in E_2E__*ndo*_ and E_2E__*xo*_ effects. Therefore, we stand with Zhang et al. (2021) in suggesting that E_2_RT should be given at dosages that mimic the concentrations of the estrous cycle to eliminate the observed variations in its cardioprotection efficacy. This will enhance the exploitation of the therapeutic potentials of E_2_RT in attenuation/prevention of CSC *via* immunomodulation in postmenopausal women.

## Data Availability Statement

The original contributions presented in the study are included in the article/supplementary material, further inquiries can be directed to the corresponding author/s.

## Ethics Statement

The animal study was reviewed and approved by the Experimental Animal Centre of Xuzhou Medical University and the Animal Ethics Committee of Xuzhou Medical University (permit no: xz11-12540).

## Author Contributions

HH conceived the experiment idea. HS, HH, GKA, and QW designed the experiments. HH and GKA isolated and cultured PM_ϕ_. HH, TM, and YM made animal models. HH, JG, MS, and LF performed cardiac function and histological assessments. HH, GKA, QW, and HS analyzed and interpreted the results. Based on the contributions of all authors, HH drafted the initial manuscript and GKA revised it entirely. HH, GKA, QW, TM, YM, JG, MS, LF, RR, and ZG proofread and approved the manuscript in its current form.

## Conflict of Interest

The authors declare that the research was conducted in the absence of any commercial or financial relationships that could be construed as a potential conflict of interest.

## Publisher’s Note

All claims expressed in this article are solely those of the authors and do not necessarily represent those of their affiliated organizations, or those of the publisher, the editors and the reviewers. Any product that may be evaluated in this article, or claim that may be made by its manufacturer, is not guaranteed or endorsed by the publisher.
